# Annotations

**Published:** 1890-10-04

**Authors:** 


### Annotations.

A Wife to Wives.

"A Wife" sends a very energetic contribution to the *Daily News* holiday discussion on "Matrons and Maids." She speaks plain truths with a matter-of-fact directness which no "husband" would think of imitating. Probably, however, a good many husbands will be secretly or openly grateful for her valiant words. "There are two sides to every bargain," says this business-like dame. "Many men marry women who have not a shilling to call their own. They work for them early and late. They buy and sell, travel, dig and delve in order to support the home or to surround the wife with every comfort. . . . As the wife in too many cases brings no grist to the mill, and for the life of her could not earn anything, it behoves her to look at the case sensibly. She should strive to make the best of things. . . . A woman can do anything if she sets her mind that way; and if only she will devote a few hours each day to the divine drudgery of the household." Now no man would remind his wife that she brought no grist to the mill; nor does he wish her to be reminded by other people. Besides, it was not her fault if she came empty-handed. She was not old enough when she married to have earned and saved a fortune. Her want of "grist" her father was responsible for, not herself. She would have been only too glad to have brought a whole cart-load. But though a husband would not remind his wife of this, there is no reason why she should not remind herself. Not that she need be the least unhappy about it. But it should be an incentive to her to regard her housekeeping as an important part of the business of "husband and wife." The majority of men make only modest incomes; and in all these cases the competence that ought to be ready for old age depends more upon the wife's savings than upon the husband's earnings. Few women are thrifty by nature, and we do not blame them much for that. A woman who is greatly given to saving is generally a thin-souled and unlovely creature. The present writer would never marry one who was the least given to miserliness. But all the same, a wife should learn economy and skilful domestic management as an art. The devil will tempt her to imitate her richer neighbours in every way; to keep four servants when three or even two might do; to be constantly changing her dressmaker and milliner, and always going to one a little more expensive than the last. The devil that offers temptations like these should be resisted, tooth and nail. You are very pleasant and agreeable, ladies, and every well-conditioned man would prefer to have an income of thousands a-year rather than of hundreds to lay at your feet. But it cannot always be! And, therefore, you should listen to such women as the "wife" who talks so sensibly in the *Daily News*. You should not wait for your husbands to scold you into economy, or to entreat you into good management. You should willingly and cheerfully take up your burden of domestic cares, and resolve that however skilful and successful your husband may be at his business, you will exceed him altogether in the economy and efficiency of your household affairs.

A Perennial Question.

Last week an experienced and honoured lady philanthropist, who signed herself "L," made one more effort in our pages to arrest the attention of the public on behalf of the out-patient question. This lady points out that provident dispensaries charge such low rates for treatment and medicine that practically all persons who are not actually destitute may afford to pay them. For those who are destitute the poor law provides excellent dispensaries in all the Metropolitan unions. "L" asks if "all or nearly all those who plead inability to pay for medical treatment do not spend far more than sixpence per month in beer or other unnecessary stimulants? Do not the children of the poorest get pence to spend in sweets and ices throughout the week; and why, if thrift were thought of, and not wholly discouraged by our present plans, should not some at least of all this expenditure be bestowed on providing for the certain and inevitable misfortunes of sickness? The answer is clear; because by our plans for past generations no inducement is given for any such expenditure, and is directly discouraged by all our treatment of the poor. . . . The 'indifference' of the poor can never be overcome so long as they are subjected to the demoralising and pauperising influences of free medical attendance, of which even the well-to-do and those in the public service do not scruple to avail themselves." . . . "L" adds in a postscript "that it is a fact that the children of the very poor spend 2s. or 3s. when taken out for a school treat." Persons who know the secrets of the out-patient departments of most of the London hospitals can endorse practically every word which "L" says. Those departments are manufactories for beggars and paupers. It is well known why the out-patient system is continued in its present form. It gives positions and appointments to many young medical men who would otherwise have no chance of belonging to the staff of a hospital. No doubt, also, it furnishes materials for the practical instruction of medical students. Those materials could, however, easily be found elsewhere if the out-patient departments of the hospitals were closed. The true inwardness of the business is that the doctors want those departments for their own convenience. This fact may be glossed over in a thousand ways; but it cannot be denied. So then it comes to this, that philanthropy is to be exploited, and tens of thousands of persons are to be annually pauperised for the sole purpose of gratifying medical ambition. This is a shameful fact; but it is a fact. If the Royal Commission does nothing to check the abuses of out-patient departments it will have failed to grapple with one of the most serious and dangerous problems of modern times.

Portobello Philosophers.

Everybody interested in the proper treatment of the sick, and in the study of certain phases of Scottish character, should procure a copy of the *Edinburgh Evening Dispatch* of September 19th, and read the article headed "An extraordinary story of mismanagement." Portobello is a small watering-place about two miles from Edinburgh, on the Firth of Forth, and is proudly designated by its inhabitants the "Brighton of the North." Some time ago a proposal was set on foot for a fever hospital. The town, quite apart from its numerous summer visitors, is large enough to require a fever hospital of considerable size. The proposal to erect such a building was strenuously resisted by some of the more close-fisted members of the town council. But under sufficient pressure from without, the hospital was at last taken in hand, and duly built. Then arose a disturbance about the furnishing, which we need not now consider. What is of importance is the fact that the first infectious case was recently received at the hospital, and the way in which the case was dealt with. About two months ago the wife of a Glasgow working man went with several of her children to Portobello for a few days holiday. Her youngest boy, a child of four, was attacked with scarlatina, and as the town was crowded with visitors, the medical man called in ordered the case to be sent to the fever hospital. When the child got to the hospital the place was not only unfurnished but destitute even of a bed. Not only was there no bed, but there was neither nurse nor doctor. A bed having been provided, it was proposed to put the child into it without its having been previously aired, and without any fire being lighted in the ward. In the end the woman and her sick son were left without nurse or doctor as the sole tenants of the dreary hospital. For days, nay for weeks, the mother was left alone to nurse and tend her boy. Neither medicines, medical attendance, nor even proper food was provided by the Hospital Committee. It is said that a few shillingsworth of groceries were sent, but that some of them were so bad that they had to be thrown out. The poor mother had to spend about five shillings a-day in medicine and food, with the result that her little savings were soon all gone and that gaunt hunger stared her and her boy in the face. Through the kindness of a railway servant she was enabled to borrow a few shillings from a former lodger, and these she spent on her boy, barely eating enough herself to preserve her from death by starvation. At length the child recovered and the mother returned home to Glasgow. She had not been long there when she received from the town authorities of Portobello a note with a demand for four pounds for "accommodation" in the hospital. The question of the payment of this amount is still pending. Now, what can any man say of these Portobello town authorities? Does Scotland really produce such fools as these; or are they imported from a distance? The cold-blooded cruelty of the business is bad enough; but it is nothing compared with its wooden-headed imbecility. We never believed before that there were such town councillors as are described in "Mansie Wauch"; but we believe it now. Poor Portobello!

